# Ampicillin Plus Ceftriaxone Regimen against *Enterococcus faecalis* Endocarditis: A Literature Review

**DOI:** 10.3390/jcm10194594

**Published:** 2021-10-06

**Authors:** Andrea Marino, Antonio Munafò, Aldo Zagami, Manuela Ceccarelli, Rosaria Di Mauro, Giuseppina Cantarella, Renato Bernardini, Giuseppe Nunnari, Bruno Cacopardo

**Affiliations:** 1 Unit of Infectious Diseases, Department of Clinical and Experimental Medicine, ARNAS Garibaldi Nesima Hospital, University of Catania, 95123 Catania, Italy; andreamarino9103@gmail.com (A.M.); aldozagami@alice.it (A.Z.); manuela.ceccarelli86@gmail.com (M.C.); cacopard@unict.it (B.C.); 2 Unit of Infectious Diseases, Department of Clinical and Experimental Medicine, University of Messina, 98100 Messina, Italy; giuseppe.nunnari@unime.it; 3 Department of Biomedical and Biotechnological Science, School of Medicine, University of Catania, 95123 Catania, Italy; uni315775@studium.unict.it (A.M.); rosariadimauro81@gmail.com (R.D.M.); gcantare@unict.it (G.C.); 4 Unit of Clinical Toxicology, Policlinico G. Rodolico, School of Medicine, University of Catania, 95123 Catania, Italy

**Keywords:** infective endocarditis, *Enterococcus faecalis*, double beta-lactams therapy, ampicillin plus ceftriaxone, antibiotic resistance

## Abstract

*Enterococcus faecalis* infective endocarditis (EFIE) continues to represent a potentially fatal infectious disease characterized by elevated morbidity and mortality. Despite advances in antimicrobial therapy, changing demographics and the reduced availability of useful antibiotics combined with the dissemination of multi-drug resistant strains, the mortality rate remained unchanged in the last decades. Nowadays, optimizing the antibiotic regimen is still of paramount importance. Historically, aminoglycosides were considered as a cornerstone for treatment even though their use is associated with a high risk of kidney failure. It is against this background that, in recent years, several studies have been carried in order to assess the validity of alternative therapeutic approaches, including combinations of beta-lactams, that, acting synergistically, have yielded useful results in different clinical settings. In this scenario, we searched and critically report clinical studies assessing the efficacy and safety of double beta-lactam therapy in treating EFIE.

## 1. Introduction

Despite major clinical advances in antimicrobial therapy, the prevalence and associated mortality of infective endocarditis (IE) have not markedly improved over the last several decades, especially those caused by fastidious germs such as Enterococci.

Enterococci are Gram positive facultatively anaerobic bacteria that can usually be seen as cocci in short chains, colonizing the human gastrointestinal (GI) tract [1,2]. *Enterococcus faecalis* and *E. faecium* are the most clinically relevant species and they represent the most common organisms, causing both hospital-associated and community-associated IE [3].

In particular, *Enterococcus faecalis* infective endocarditis (EFIE), accounting for the third frequent cause of both native and prosthetic valve IE in the community setting and the second cause of healthcare-associated infective endocarditis (HAIES), still poses major clinical and therapeutical issues [4,5]. Bacteremia and IE are common presentations of enterococcal disease, and the most frequent sources of bacteremia are the GI and genitourinary tracts among non-hospitalized patients, whereas urinary and intravascular catheters are the most common sources of nosocomial bacteremia, especially in those who have received antibiotics or with underlying conditions. Urinary tract infections and diagnostic/therapeutic instrumentation (such as urinary catheter, cystoscopy, prostatic biopsy and transurethral resection of the bladder or prostate) are potential causes of EFIE and the same risk factors are applied to the GI tract [1]. Moreover, *E. faecalis* is one of the main causative microorganisms of transcatheter aortic valve implantation-associated endocarditis (TAVIE), an emerging and poorly characterized infection marked by high mortality that is becoming prevalent with the increasing number of TAVI procedures performed in recent years [6]. Notably, *E. faecalis* seems to be the most frequent detected agent in TAVIEs diagnosed within two months after TAVI [7].

EFIE usually involves damaged heart valves and mitral and aortic valves are usually interested. Most patients with EFIE display a subacute course and the most common complication is heart failure occurring in about half of patients, with a significant percentage requiring valve replacement [8].

Furthermore, on account of *E. faecalis* being established as a common cause of HAIES, considering that enterococci are naturally tolerant to a number of antimicrobial compounds, this disease is becoming increasingly prevalent among the elderly and patients with relevant comorbidities, making the treatment of this infection particularly troublesome and contributing to the unchanged mortality rates [3,9,10].

This worrying picture is further worsened by the usual lack of reliable bactericidal activity of most antimicrobials and by the renowned nephrotoxicity arising from the synergistic therapeutic combination of beta-lactams plus aminoglycosides, which, from the 1950s, has been indisputably recognized as treatment of choice for EFIE [11]. However, the widespread development of high-level aminoglycosides resistance (HLAR) strains [12], whichever the mechanism, abolished the synergism of the combination with cell wall agents, reducing the likelihood of obtaining a favorable clinical outcome [13]. This scenario, coupled with the abovementioned toxicity issues related to prolonged aminoglycosides administration, has prompted efforts to identify different pharmaceutical effective solutions for the treatment of EFIE [14].

With this purpose, experimental studies have been successfully performed in order to assess the synergism of the dual beta-lactam combination against clinical strains of *E. faecalis*, regardless of their susceptibility to aminoglycosides. Specifically, the basis for the synergistic activity of the double beta-lactam combination appears to be related to the differential and complementary saturation of *E. faecalis* penicillin-binding proteins (PBPs), thus generating the necessary bactericidal effect [15,16]. Thereafter, these experimental results were supported by clinical evidence, leading to an update of EFIE treatment guidelines [17,18].

## 2. An Unmet Need

Aminoglycoside-based regimens have been a cornerstone of antimicrobial therapy for EFIE and have been recommended as therapy of choice for decades. However, the effectiveness and safety of this therapeutic approach have been threatened by the increasing acknowledgement of aminoglycoside-resistant strains. Additionally, bearing in mind that the typical EFIE patient is older, often debilitated, with high rates of chronic renal failure and/or with enhanced risk of rapid renal impairment, the standard 4-to-6-week course of aminoglycoside therapy could result in serious, possibly life-threatening, nephrotoxic complications. It is against this background that, in recent years, a number of studies have been carried out in order to assess the validity of alternative therapeutic approaches, including a treatment regimen with different dosage and duration of aminoglycoside administration, with the main purpose of reducing the known toxicity. Moreover, not all clinical laboratories may have the capability for rapid determination of serum gentamicin concentrations available to assist in optimal dosing adjustments. These factors, as a whole, have prompted studies to evaluate the efficacy of non-aminoglycoside-containing regimens for the treatment of EFIE [19,20,21].

## 3. Preclinical Evidence for an Alternative Therapeutic Approach

In light of the few therapeutic alternatives, combinations of beta-lactams were tested in vitro and in vivo models of enterococcal experimental IE.

First, Mainardi and colleagues [22] reported in vitro synergy between amoxicillin and cefotaxime against clinical strains of *E. faecalis*, showing that the minimum inhibitory concentration (MIC) for amoxicillin decreased substantially in the presence of cefotaxime, and likewise, MIC of cefotaxime in the presence of amoxicillin. This effect was explained by the differential targeting of the PBPs by each beta-lactam compound, which, combined, cause a significant inactivation of PBPs 2, 3, 4 and 5, producing a marked impairment in *E. faecalis* cell wall synthesis [23]. This in vitro synergy was recently confirmed by Liao et al., demonstrating on time-kill curves a reduction in the number of colony-forming units (CFU) of *E. faecalis* after exposure to ampicillin and ceftriaxone when compared with those exposed only to ampicillin [24]. In addition, it was further corroborated in an in vitro pharmacodynamic study in which ampicillin-cephalosporin combinations showed an increased activity compared to ampicillin alone against both strains of *E. faecalis* (ampicillin-susceptible gentamicin-susceptible strain (OG1X) and HLAR strain (HH22) over 24 h [25].

Similar synergistic findings were detected by Gavalda et al. [14] with the association of ampicillin and ceftriaxone against HLAR *E. faecalis* strains. These results have suggested that bactericidal ampicillin concentrations moved into the bactericidal ones by association with ceftriaxone, indeed extending the range of ampicillin’s bactericidal effects and the period during which these concentrations are available. Along this line, the same authors have evaluated the usefulness of ceftriaxone combined with ampicillin, compared to ampicillin plus gentamicin, against *E. faecalis* with or without HLAR in rabbits with catheter-induced endocarditis, concluding that was effective as the treatment of choice [26]. Recently, in addition, according to a multiple antibiotic dosing scheme based on the half-lives of one of the tested antibiotics, the ampicillin and ceftriaxone combination exhibited synergistic interactions against *E. faecalis* in the *Galleria melonella* infection model [27].

Nevertheless, further studies are needed to achieve a more accurate estimation of pharmacodynamic parameters and the determination of the pharmacokinetic/pharmacodynamic (PK/PD) index, driving the efficacy of this combination, essential for a proper application in clinical practice.

## 4. A Critical Analysis of the Clinical Experience of This Therapeutic Alternative

These experimental results laid the groundwork for clinical studies aimed at establishing the true efficacy of this therapeutic approach in humans with EFIE.

The first one, conducted by Gavalda et al. [28] was an observational, multicenter, open-label clinical trial designed to evaluate the efficacy and the safety of treatment with ampicillin, 2 g every 4 h, plus ceftriaxone, 2 g every 12 h, as an antimicrobial option in patients with endocarditis caused by *E. faecalis* with or without HLAR. The clinical cure, defined as the resolution of the clinical findings of endocarditis with no evidence of active endocarditis at both the end of treatment and 3-month follow-up visit, achieved the rate of 67.4% (29 of 43 patients) among all patients. The treatment-related mortality rate of patients with HLAR EFIE was 28,6%, similar to rates reported in previous studies. With regard to adverse events, the double beta-lactam combination was well tolerated and only two patients had treatment-related side effects and no case of nephrotoxicity was recorded.

Notwithstanding the substantial limitations given by the small size of the sample, the lack of a random assignment and the delayed inclusion of patients with non-HLAR EFIE, the study provided significant results supporting the employment of double beta-lactam combination as an effective treatment for patients with HLAIR EFIE and, in addition, as a wise option for patients with high risk of nephrotoxicity, regardless of strain susceptibility.

These compelling results provided the rationale for a large, non-randomized, non-blinded, comparative, multicenter cohort study conducted by Fernandez-Hidalgo and colleagues [29]. The aim was to assess the safety and efficacy of the ampicillin-ceftriaxone combination in the treatment of EFIE compared with the standard of care antimicrobials, ampicillin plus an aminoglycoside. Notably, this study included 159 patients treated with ampicillin plus ceftriaxone (A+C) and 87 treated with ampicillin plus gentamicin (A+G). The authors concluded that, even though A+C treated patients were in poorer general condition before acquiring the infection than A+G patients, no differences were found between the two treatment arms in mortality, during treatment or at 3 months of follow-up, in clinical failure (i.e., new vegetation, septic paravalvular complications or persistently positive blood cultures) and in relapse (defined as positive blood culture with initial pathogen during follow-up) rates. However, a higher proportion of A+G patients switched or stopped gentamicin owing to renal failure, not receiving, therefore, the complete course of the aminoglycoside-containing regimen. Although the overarching results of the current study are striking, several caveats need to be taken into consideration to properly interpret the reported data. First, this study was designed as a superiority trial but aimed to demonstrate non-inferiority. Moreover, it is unclear whether the trial had sufficient power to detect a significant difference between the two treatment regimens. In addition, despite the wide experience of all the participating centers in managing IE patients and the prospective method of data acquisition, most cases were retrospectively collected. Furthermore, although comparative, this study was not randomized and treatment recommendations depended on the one hand, on center discretion and, on the other, based upon the baseline renal function and/or the risk of new renal failure. Thus, unmeasured confounding factors as well as selection bias cannot be ruled out. In addition, the definition of acute renal failure, established as a 25% increase in the baseline creatinine concentration, was a rather liberal and misleading extent that could have overestimated the renal dysfunction rate. The assessment of glomerular filtration rate and/or the use of the RIFLE score would have been more appropriate. Consequently, the interruption of aminoglycoside therapy was left to physicians’ choice, thus introducing further biases in the study evaluation. Moreover, among the 87 patients in the A+G group, dose scheduling was variable, including the recommended regimen (thrice daily) only for 37 patients, whereas an additional 37 patients received a once-daily regimen and the remaining ones received gentamicin twice daily. Unfortunately, information regarding gentamicin levels were reported only for 60% of patients and no analysis of outcomes based on dose schedule was performed, allowing a broad discretion in the prescription or not of alternative agents; such factors could have adversely influenced the efficacy or safety evaluation of the regimen. Nonetheless, although these considerations highlight the difficulties encountered when treating EFIE, this study provided useful clinical data in a field in which information and therapeutic alternatives are limited, supporting the use of A+C for the treatment IE caused by *E. faecalis.*

With the aim of shedding light on this challenging issue, Pericas et al. [30] performed a monocenter retrospective analysis of a prospective cohort of EFIE patients treated from 1997 to 2011, with the objective to assess resistance patterns, epidemiology and clinical outcomes. Interestingly, through the collection and analysis of epidemiological data, the authors detected an overwhelming increase in EFIE caused by HLAR strains over the course of the last years, along with an increase in the use of A+C therapy. Although the statistical power of results is limited by the small sample size, these data appear meaningful, indeed presenting a similar trend to the most recent reported relapse rates. Furthermore, similarly to Fernandez-Hidalgo and colleagues, the authors did not detect a significant difference in in-hospital mortality (27% vs. 23%), 1-year mortality (29% vs. 26%) and relapse rate (2 vs. 3) between patients respectively treated with A+G and those treated with A+C. Despite the survival and regression analyses showing no statistical difference in 1-year mortality between the two treatments, these parameters cannot be used to conclude that there are no clinical differences between groups because the study was not powered to detect this. Of note, patients who received A+G presented a higher incidence of renal failure during treatment, requiring a therapeutic switch to A+C and further influencing the results.

These studies supported an update in the most recent American Heart Association IE treatment guidelines [17], together with the guidelines of the European Society of Cardiology [18], that recommended the use of the A+C combination as a treatment option for EFIE in patients with HLAR strains and as a reasonable alternative in those with impaired renal function and/or at high risk to develop nephrotoxicity due to aminoglycoside therapy, regardless of HLAR status.

Moreover, another retrospective cohort study conducted by El Rafei and colleagues [31] further sustained previous results, supporting double beta-lactam combination as a safe alternative to A+G for treating EFIE, regardless of aminoglycoside susceptibility. In addition, a recent prospective multicenter cohort study was addressed to compare the efficacy of a shorter course of A+C (4 weeks) with respect to the recommended duration (6 weeks) for the treatment of native valve EFIE. Despite the statistical power being significantly limited by the small size of the sample and by the lack of randomization, this study reported similar rates of relapse and mortality between treatment groups. Thus, suggesting that a shorter treatment course might represent an alternative regimen, notably in patients with a briefer duration of symptoms and those without perivalvular abscess. Even in this case, further research is required to validate these results [32].

Overall, the clinical data, which evaluated the usefulness of A+C regime, are summarized in Table 1.

Although the aminoglycoside-containing regimen has been the standard of EFIE treatment, the worrying rise in resistance and the availability of less nephrotoxic agents has led to fine-tuning a novel treatment option. The former studies have assessed the safety and efficacy of the double beta-lactam combination for EFIE and suggest A+C combination as a therapeutically similar option with lower rates of treatment discontinuation. However, whilst they have made important contributions, each one had significant limitations, above all, the variability in selection of treatment regimen due to the lack of a randomized assignment. Moreover, their retrospective nature with limited case numbers further underlines the need to perform a randomized clinical trial (RCT) with the purpose to answer whether the double beta-lactam combination is preferable to A+G in terms of cure, relapse rate, survival and drug-related adverse events. Nevertheless, it is worth emphasizing that, due to the specific features of EFIE patients and the low prevalence of the disease, a proper enrolment in a RCT becomes fairly unfeasible [33].

## 5. Conclusions

The decision to use an aminoglycoside-containing regimen must be individualized for each patient. The recommendation for a specific aminoglycoside-containing therapy should not be exclusively based on in vitro susceptibilities, but several factors must be considered. In general, patients with EFIE are often debilitated and have significant underlying comorbidities common in older age groups, hence the well-known gentamicin-associated nephrotoxicity and ototoxicity of a standard 4-to-6-week course of therapy could result in serious complications with the concrete likelihood of subverting the positive benefit/risk ratio. Although the reported clinical studies assessing the validity of double beta-lactam therapy compared with ampicillin plus gentamicin regimen in treating EFIE were observational, largely retrospective and non-randomized, these provided important data. Above all, similar success and mortality rates combined with the lower risk of nephrotoxicity and the lack of need for measuring aminoglycoside serum concentrations, place this therapeutic combination as a meaningful and wise treatment option for patients with EFIE regardless of HLAR status. Therefore, patients receiving this therapy should be monitored for leukopenia by assessing complete blood counts weekly after initiation of treatment and for the increased risk of vancomycin-resistant *Enterococcus* (VRE) gastrointestinal colonization [34,35].

Overall, the epidemiological changes of EFIE, with ageing and frail populations and an underestimation of treatment side-effects, namely the high risk of nephrotoxicity, should force a paradigm shift in the antibiotic choice. Although the current data are not definitive, the growing body of literature with the combination ampicillin and ceftriaxone appears promising. For this purpose, large and high-quality non-inferiority clinical studies, even if prospective and not randomized, are needed to definitively assess the efficacy and safety of double beta-lactams regimes against EFIE.

## Figures and Tables

**Table 1:** Clinical data evaluating dual beta-lactam combination therapy in EFIE treatment (A = ampicillin; C = ceftriaxone; G = gentamicin; * = completed therapy).

Reference	Study Design	Subjects	Dose Regimen	Follow-Up	Mortality %	Renal Impairment %	Relapses%	Main Finding
Gavaldà et al. (2007) [28]	Observational, open label, non-randomized, multicenter clinical trial observing outcomes in patients receiving ampicillin plus ceftriaxone treatment.	43 patients with EFIE	A 2g q4h + C 2g q12h for 42 days (5–48)	3 months	Overall 28%	No cases occured	4.6%	The combination of ampicillin and ceftriaxone is effective and safe for treating HLAR EFIE and could be a reasonable alternative for patients with non-HLAR EFIE who are at increased risk for nephrotoxicity.
Fernández-Hidalgo et al. (2013) [29]	Non-randomized, non-blinded, comparative, multicenter cohort study comparing ampicillin plus ceftriaxone and ampicillin plus gentamicin in patients with endocarditis.	246 patients with EFIE	A 2g q4h + C 2g q12h [(A+C) *n* = 159] vs. A 2g q4h + G 3 mg/kg/d for 4–6 weeks [(A+G) *n* = 87]	11 months (4.4–22.5 months)	Overall 26 % (A+C) Overall 25% (A+G)	33% (A+C) 46% (A+G)	3% (A+C) 4% (A+G)	Ampicillin plus ceftriaxone appears as effective as ampicillin plus gentamicin for treating EFIE patients and can be used with virtually no risk of renal failure and regardless of the HLAR status.
Pericas et al. (2014) [30]	Retrospective analysis of prospectively collected data assessing antibiotic resistance, epidemiology and comparing safety and efficacy of ampicillin plus ceftriaxone and ampicillin plus gentamicin in patients with endocarditis.	69 patients with EFIE	A 2g q4h + C 2g q12h [(A+C) *n* = 39] vs. A 2g q4h + G 3 mg/kg/d for 4–6 weeks [(A+G) *n* = 30]	13 months (118–792 days)	1 year 26% (A+C) 1-year 30% (A+G)	34% (A+C) 65% (A+G)	8% (A+C) 3% (A+G)	The prevalence of HLAR EFIE has increased significantly in recent years and that alternative treatment with ampicillin and ceftriaxone is safer than ampicillin plus gentamicin, with similar clinical outcomes.
El Rafei et al. (2018) [31]	Retrospective cohort study comparing safety and efficacy of dual β-lactam therapy to penicillin-aminoglycoside combination in patients with endocarditis	85 patients with EFIE	A 2g q4h + C 2g q12h [(A+C) *n* = 18] vs. A 2g q4h + G 3 mg/kg/d for 4–6 weeks [(A+G) *n* = 67]	12 months	1-year 11% (A+C *n* = 13) * 1-year 9% (A+G *n* = 37) *	11% (A+C) 25% (A+G)	7.7 % (A+C) * 2.7 % (A+G) *	Ampicillin plus ceftriaxone appears to be a safe and efficacious regimen in the treatment of EFIE. Patients treated with this regimen had lower rates of nephrotoxicity and no differences in relapse rate and 1-year mortality as compared to that of the ampicillin plus gentamicin group.
Ramos-Martinez et al. (2020) [32]	Prospective non-randomized cohort study comparing the efficacy of shorter courses of AC (4 weeks) with respect to the recommended duration of 6 weeks for the treatment of EFIE.	109 patients with EFIE	A 2g q4h + C 2g q12h for 28 ± 4 days [(4 weeks) *n* = 39] vs. A 2g q4h + C 2g q12h for 42 ± 6 days [(6 weeks) *n* = 70]	12 months	1-year 17% (4 weeks)1-year 21.4 % (6 weeks)	25.6% (4 weeks) 28.6% (6 weeks)	5.1 % (4 weeks) 4.3 % (6 weeks)	Similar rates of relapse and mortality were recorded in patients with native valve EFIE treated with A+C for 4 and 6 weeks, suggesting that a short course of A+C might be sufficient to treat native valve EFIE.

## Data Availability

No new data were created or analyzed in this study. Data sharing is not applicable to this article.
